# Advances in physical and chemical strategies for dentin hypersensitivity therapy

**DOI:** 10.1016/j.jare.2025.09.041

**Published:** 2025-09-23

**Authors:** Xinru Li, Qihui Wang, Yirong Sun, Guoliang Wang, Congxiao Zhang, Jianxun Ding

**Affiliations:** a Department of Stomatology, The First Hospital of Jilin University, 1 Xinmin Street, Changchun 130061, PR China; b State Key Laboratory of Polymer Science and Technology, Changchun Institute of Applied Chemistry, Chinese Academy of Sciences, 5625 Renmin Street, Changchun 130022, PR China; c School of Applied Chemistry and Engineering, University of Science and Technology of China, 96 Jinzhai Road, Hefei 230026, PR China

**Keywords:** Laser treatment, Biomimetic mineralization, Biomaterial, Synergistic strategy, Dentin hypersensitivity therapy

## Abstract

**Background:**

Dentin hypersensitivity (DH) is a prevalent and acute dental pain disorder marked by a brief yet sharp toothache caused by exposed dentin, often in response to thermal, chemical, and mechanical stimuli. It significantly affects a person's quality of life, interfering with routine activities, such as eating and drinking. With an improved understanding of the mechanisms underlying DH, various physical and chemical strategies have been developed to alleviate the condition.

**Aim of the review:**

This review evaluates three principal mechanistic theories underlying DH desensitization, including the Direct Innervation Hypothesis, the Odontoblast Receptor Theory, and the Hydrodynamic Theory. It provides a detailed analysis of current physical, chemical, and synergistic therapeutic methods used for desensitization. Additionally, it discusses potential future therapeutic approaches for the treatments of DH.

**Key scientific concepts of review:**

This review highlights recent advancements and ongoing challenges in applying physical and chemical strategies for DH therapy. It also discusses potential future strategies and methods, offering insights into the prospects for the field.

## Highlights

• Dentin hypersensitivity is characterized by pain resulting from the exposure of dentinal tubules.
• Sealing exposed dentinal tubules is a key strategy to alleviate dentin hypersensitivity.
• Physical, chemical, and synergistic approaches are employed in desensitization therapies.

## Graphical abstract

**Figure:**
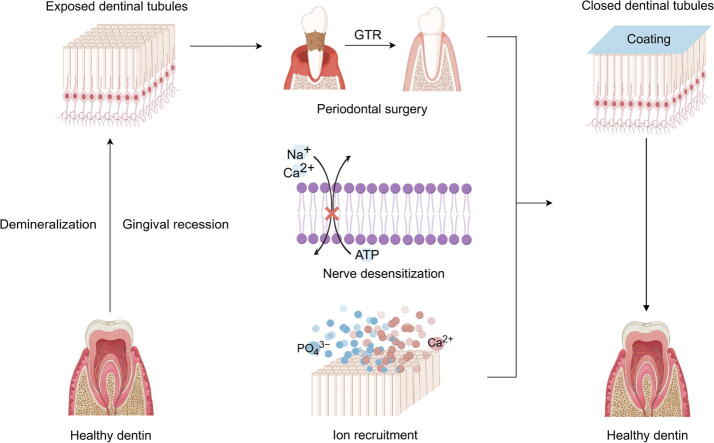

## Introduction

Dentin hypersensitivity (DH) is a prevalent oral disorder clinically manifested by brief and acute toothache triggered by various exogenous stimuli acting on exposed dentinal tubules, including thermal changes, chemical substances, and mechanical action [1]. In severe cases, this sensitivity may disturb the patient's routine activities, such as eating, drinking, brushing, and breathing [2]. A meta-analysis of studies conducted in various countries and populations estimated the average prevalence of DH at 33.5 %, depending on the analysis model [3].

**Scheme 1:**
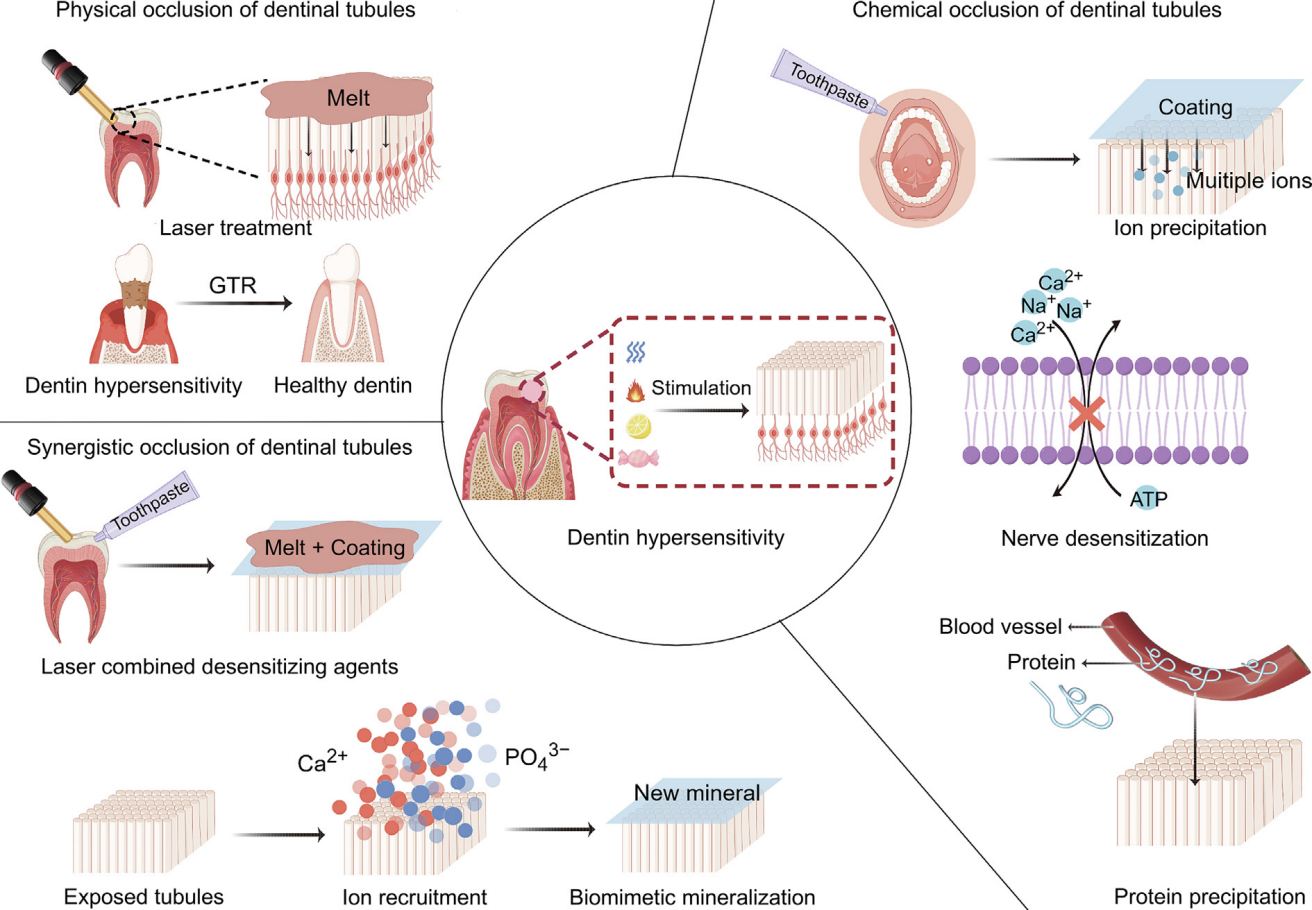
Various desensitization treatments for DH. Created by Figdraw. X. Li, (2025), figdraw.com/WRIRAa0aab.

Many factors contribute to the onset of DH, including gingival recession exposing the dentin, loss of enamel or cementum, dental erosion and abrasion, plaque accumulation, and bleaching [4]. In addition, the prevalence of DH may be higher among individuals with tooth misalignment (crowding or labial-side flipped teeth), poor habits, and after orthodontic or periodontal treatment compared to the general population [5,6]. Orthodontic tooth movement that shifts teeth outside the buccal plate increases the risk of gingival recession, which subsequently causes DH [7]. Periodontal treatment may lead to more pronounced gingival recession and increased exposure to root surface. The prevalence of DH or root hypersensitivity was between 76.8 % and 80.4 % within one day after periodontal surgical therapy [8]. Simultaneously, in a *in vitro* study (60 dentine samples), suggested that a higher brushing force (400 g) lead more tubules were exposed [9]. A case-control study on 600 participants indicated that contact time between the tooth and acid is the risk factor of DH [10]. Due to extensive research on the contributing factors of DH, its persistently high prevalence underscores the urgent need to understand DH's pathogenesis to treat it effectively (Scheme 1).

Multiple hypotheses have been proposed to explain DH development. A leading proposed mechanism, the “Direct Innervation Theory”, states that nerve fibers extend into dentinal tubules, with some terminal branches approaching the dentin-enamel junction (DEJ) [11]. External stimuli act directly on the nerves (A − β fibers and minor A − δ subpopulations), generating the prototypical brief, lancinating toothache associated with DH [12]. However, this direct innervation has been observed to extend only for a minimal distance into the inner dentin [4,13]. Later theory, “Odontoblast Receptor Theory”, proposes that odontoblasts function as mechanosensory cells, converting external stimuli into the nerve terminal, which in turn generates the sensation of toothache from the nerve endings situated at the pulp-dentinal border. Odontoblast processes extend only into the proximal 1/3 of dentinal tubules and cannot reach the DEJ, where processes degenerate with age, and hypersensitivity symptoms persist [4]. The most currently favored theory proposes that the sensation of toothache resulting from external stimuli is the “Hydrodynamic Theory”, initially proposed by Brannstrom *et al.* This theory suggests that external stimuli cause fluid movement inside dentinal tubules, which activates pulp nerve fibers, resulting in the toothache sensation [11,14].

A recent study demonstrated that directional cation transport through the dentinal tubules is also a mechanism of DH. The dentinal tubules present an asymmetrically charged microenvironment, and the negative potential increases from the distal to proximal end of the dentinal tubules. External stimuli, such as cold, hot, sour, and sweet, trigger the directional movement of cations in the dental canal by affecting ion flow rate, ion diffusion coefficient, ion concentration gradient, and other parameters, thereby converting the stimulus into current and efficiently transmits sensory signals to stimulate the pulp nerve to produce action potentials, resulting in sensitive toothache symptoms [15,16].

Another study has shown that the dentinal tubules in DH are wider (2 × ) and more numerous (8 × ) than in non-sensitive dentin, which increases the area of contact between external stimuli and the dental pulp, thereby increasing the risk of toothache [17]. Thus, closing the exposed dentinal tubules and blocking the pulpal nerve signal transduction are significant for DH management.

The management of DH can be generally divided into two different approaches: Physical and chemical strategies (Table 1). Physical treatments include laser desensitization and restorative treatments. Low-level laser therapy, such as helium−neon (He−Ne) laser, affects neural transmission, while medium-power lasers, including diode laser, irradiate the dentin surface, causing the occlusion or narrowing of exposed dentinal tubules [18,19]. Restorative treatments, including direct resin−matrix composite or glass ion-based repair, indirect crown repair, veneer repair, or surgical correction of gingival recession, achieve effective and long-lasting desensitizing effects.

**Table 1:** Different strategies and clinical products used for treating DH.

Strategy	Clinical product	Component	Mechanism and characteristic	Ref.
Physical occlusion	Laser	Nd:YAG laser	Seal dentinal tubules by melting and re-crystallization of dentin. When the power was more than 1.5 W, dentin protein could be seen and pulp may be injured.	[96]
		Er:YAG and GaAlAs lasers	Mediate an analgesic effect related to the depolarization of C-fiber afferents rapid and effective clinical results.	[72,97]
		Er,Cr:YSGG laser	Seal through the vaporization of intratubular dentinal fluid, showing an immediate dentine desensitization effect.	[98]
Chemical occlusion	Toothpaste	Sensodyne® (KNO_3_)	Increase the extracellular K^+^ concentration, consequently depolarizing the nerve and preventing it from repolarizing.	[99,100]
		NovaMin® (Calcium sodium phosphosilicate, CSPS)	NovaMin® adhered to an exposed dentin surface and reacts with it to form a mineralized layer. The formed layer was resistant to acid challenge and mechanically strong.	[55]
		BioMin F® (A bioactive glass incorporate fluoride)	BioMin F® developed hydroxyapatite on the tooth surface, blocking exposed dentinal tubules. providing much longer-term resistance to dentine hypersensitivity.	[101]
	Desensitizing agent	Gluma® (Glutaraldehyde)	Glutaraldehyde reacted with serum albumin in the tubular fluid, which induced the serum albumin deposition. The effects of this agents were expected to gradually decrease with the wear caused by tooth brushing and foods.	[102]
		Bifluorid 12 (Fluoride varnish)	It created a barrier by precipitating calcium fluoride (CaF) on the dentin surface and caused occlusion of the dentin tubules.	[103]
Synergistic occlusion	tooth-desensitizing gel	Amorphous calcium magnesium phosphate (ACMP)	ACMP particles induced mineralization on the dentin surface and tubules by deep penetration of the particles and rapid release of Ca^2+^, magnesium ion (Mn^2+^), and PO_4_^3−^ that elevated pH and crystallize into hydroxyapatite, with good adherence of the occluding material and the tubule walls.	[104]
		BAG and laser	Combining bioactive glass with laser enhanced the obliteration of exposed dentinal tubules and triggered a regenerative response from dental pulp stem cells.	[71]
		K-doped bioactive glass	Combine mechanical occlusion with neural desensitization.	[88]

Chemical treatments utilize various desensitizing agents to alleviate toothache. The desensitizing kinds of toothpaste (*e.g.*, Sensodyne®, Novamin®, Biomin F®, and GARDA SILK) containing potassium (K) salts reduce neuronal excitability through membrane depolarization. Multiple agents achieve desensitization through tubule occlusion, strontium (Sr) salts induce mineral deposition, fluoride compounds (SnF_2_, NaF) form protective barriers, and silver formulations (AgF, AgI) combine occlusion with anti-bacterial action. Biomimetic materials, such as calcium silicate (CaSi), sodium phosphate (NaP), and arginine (Arg), create durable tubular seals [[20], [21], [22], [23]]. Nevertheless, many desensitizing agents demonstrate limited durability when subjected to oral conditions. Many of these products were shown to dissolve upon immersion in artificial saliva or to dislodge through chewing, brushing, and mechanical force [24].

This review is based on different desensitization mechanisms, both physical and chemical, highlighting a range of methods, such as laser, fixed crowns, desensitization toothpaste, and others. Furthermore, it also examines the potential benefits of combining physical and chemical strategies in DH treatment.

## Physical occlusion of dentinal tubules

Desensitization refers to reducing or eliminating tooth sensitivity, which primarily arises when the dentinal tubules are exposed. Since hypersensitive teeth exhibit a higher density of exposed dentinal tubules with increased diameters, effectively occluding dentinal tubules demonstrates clinically significant reductions in hypersensitivity symptoms [25]. Physical occlusion strategies, such as an array of different types of laser, restorative materials, and periodontal surgery, have been used to reduce the permeability of dentin [26].

### Laser desensitization

Laser plays a significant role in stomatology, offering benefits, such as enamel remineralization and anti-bacterial effects [[27], [28], [29]]. They also recrystallize the mineral component of dentin, making them useful in desensitizing treatments. Common laser types used for DH treatment include Nd:YAG, Er:YAG, Er,Cr:YSGG, and Er:YSGG lasers, carbon dioxide (CO_2_) laser, Nd:YAP laser, He Ne laser, GaAlAs laser, and so on [30]. Nd:YAG laser induces partial or completes occlusion of dentinal tubules by melting the peritubular dentin, while Er,Cr:YSGG laser contributes to tubule sealing through the vaporization of intratubular dentinal fluid. Studies have reported that the thermal effects of CO_2_ laser are highly absorbed by nano-hydroxyapatite (nHA), reducing the water content of crystallization, improving the physical properties, and stabilizing the HA of the dentin surface [31,32]. Following Nd:YAP laser irradiation also decreased the dentinal tubules' diameter and caused the subsequent alleviation of DH [33]. Laser therapy demonstrates significant efficacy in managing DH, providing immediate pain relief and sustained long-term therapeutic effects, offering superior treatment speed and greater patient acceptance [34].

Despite its advantages, laser therapy presents several limitations, laser treatment has disadvantages compared to conventional approaches, including high cost, complex use, and decreased effectiveness over time [35]. Additionally, many studies have shown that excessive temperature induces pulp necrosis, with the normal pulp temperature tolerance range being 20−50 °C, or leads to dehydration and rupture of the tooth's hard tissue [36]. Therefore, the safety of laser is worthy of further exploration.

### Restorative treatments

The dental structure consists of enamel, dentin, cementum, and pulp. Enamel, the outermost layer, is a protective shield for the underlying pulp and dentin and a barrier against external threats. Therefore, it is feasible to use restorations or periodontal surgical procedures to effectively resolve hypersensitivity by eliminating the underlying anatomical predisposing factors. Surgical periodontal treatments include guided tissue regeneration (GTR), coronal advancement flap, connective tissue grafting, free gingival grafting, and mucogingival surgery, which aim at root coverage and reduce areas of exposed dentin [37,38]. For DH caused by erosion or wear, treatments, such as direct resin−matrix composite, glass ion-based repair, and indirect crown or veneer repair, achieve effective and long-lasting results. For example, after 24 cycles of erosive challenges, a thin coating of flowable composite resin 150 μm in thickness only lost 7 % [[39], [40], [41]]. A recent meta-analysis report a decrease in cervical DH following root coverage surgery [42]. However, not enough scientific evidence to conclude that surgical root coverage procedures predictably reduce DH. Thus, further rigorous, well-conducted clinical trials are needed [37].

These physical desensitization methods are effective in blocking dentinal tubules. However, it is essential to note that laser wavelengths, power, and application distance to dentin. To ensure patients' comfort and safety, clinicians must carefully regulate the power output and duration of laser irradiation. Studies suggest that using lower powers and shorter exposures reduces risk while providing effective desensitization [43]. Sicilia *et al.* also mentioned that the application of a diode laser at a wavelength of < 780 nm and at an output power below 30 mW, with an application time of < 3 min is a safe treatment concerning pulp [44]. Furthermore, restorative procedures may induce irreversible damage to normal tooth tissue during preparation. Thus, it is recommended that non-invasive strategies be employed for 3−4 weeks for the toothache. If the pain remains unresolved, subsequently, more invasive treatment, such as periodontal surgery or tooth extraction, can be considered [45].

## Chemical occlusion of dentinal tubules

While physical interventions effectively restore lost tooth structure and provide mechanical protection for exposed dentin, they may not fully target the underlying physiological mechanisms of pain transmission. Consequently, chemical desensitization strategies have emerged as a critical complementary or alternative approach. Chemical occlusion strategies, such as ion precipitation, nerve desensitization, and protein precipitation, are significant in realizing long-term, practical desensitization effects.

### Ion precipitation

Desensitizing toothpaste is a convenient, economical, simple, and non-invasive treatment for DH. Numerous desensitizing toothpastes contain active ingredients that facilitate the occlusion of dentinal tubules by releasing bioactive ions, such as K, Sr, Ca, and phosphorus (P) [46]. Products, such as Novamin®, Sensodyne®, and Biomin F®, utilize bioactive ingredients that effectively occlude dentinal tubules and reduce sensitivity, as demonstrated in multiple clinical studies [[47], [48], [49]]. A clinical research found the symptoms of sensitivity (cold water test) reduction of 38.6 % after six weeks of using NovaMin® toothpaste [50,51]. Furthermore, 95.8 % of Sensodyne® users reported experiencing fewer DH episodes following the use of it [52]. BioMin F® bioglass particles are smaller than those found in NovaMin products exhibited a long-lasting sensitivity relief [53].

Studies demonstrate that the degree of dentinal tubule occlusion correlates positively with bioactive glass (BAG) concentration. While BAG applied directly to dentin surface exhibits poor retention and is readily dislodged by rinsing, incorporating BAG into toothpaste formulations—particularly when silica is substituted with BAG—significantly improves resistance to displacement under mechanical (brushing) and chemical (pH) challenges [54,55]. NovaMin®, a bioactive glass, releases calcium ion (Ca^2+^) and phosphate ion (PO_4_^3−^) when exposed to an aqueous medium, forming an HA-like layer chemically similar to that currently in enamel and dentin [56]. Novamin® is also included in Sensodyne® toothpaste to treat DH [57]. The strong surface affinity of these two formulations for collagen (Col) enhances dentin bonding, effectively occluding the dentinal tubules [58]. BioMin F® penetrates the dentin canal and deposits tiny BAG particles on the tooth surface. It also diffuses in saliva to form fluorapatite and releases fluorine for up to 12 h after brushing, providing long-term protection against DH [59]. Despite promising *in vitro* results, clinical evidence supporting BAG's long-term efficacy in managing DH remains limited, highlighting the need for further *in vivo* validation [60].

Polyol Germanium Complex (PGC) contains bioactive ligands (Ca, P, Ge, Mg, Zn, and threonine (Thr)) that enhance Ca metabolism and facilitate enamel-like mineralization [61]. Clinical evaluations demonstrate that PGC effectively occludes dentinal tubules, offering durable DH relief and measurable improvements in patient-reported outcomes. Venteil Group, a Russian pharmaceutical company, has developed an innovative HA-based toothpaste (GARDA SILK), including PGC, as a clinically viable, non-invasive therapeutic option for DH patients. After 14 days, the baseline Schiff sensitivity scores from 2.38 ± 0.181 decrease to 0.72 ± 0.429 [61]. However, due to their milder formulation, desensitizing toothpaste may be less effective at removing dental plaque than regular fluoride toothpaste.

**Fig. 1:**
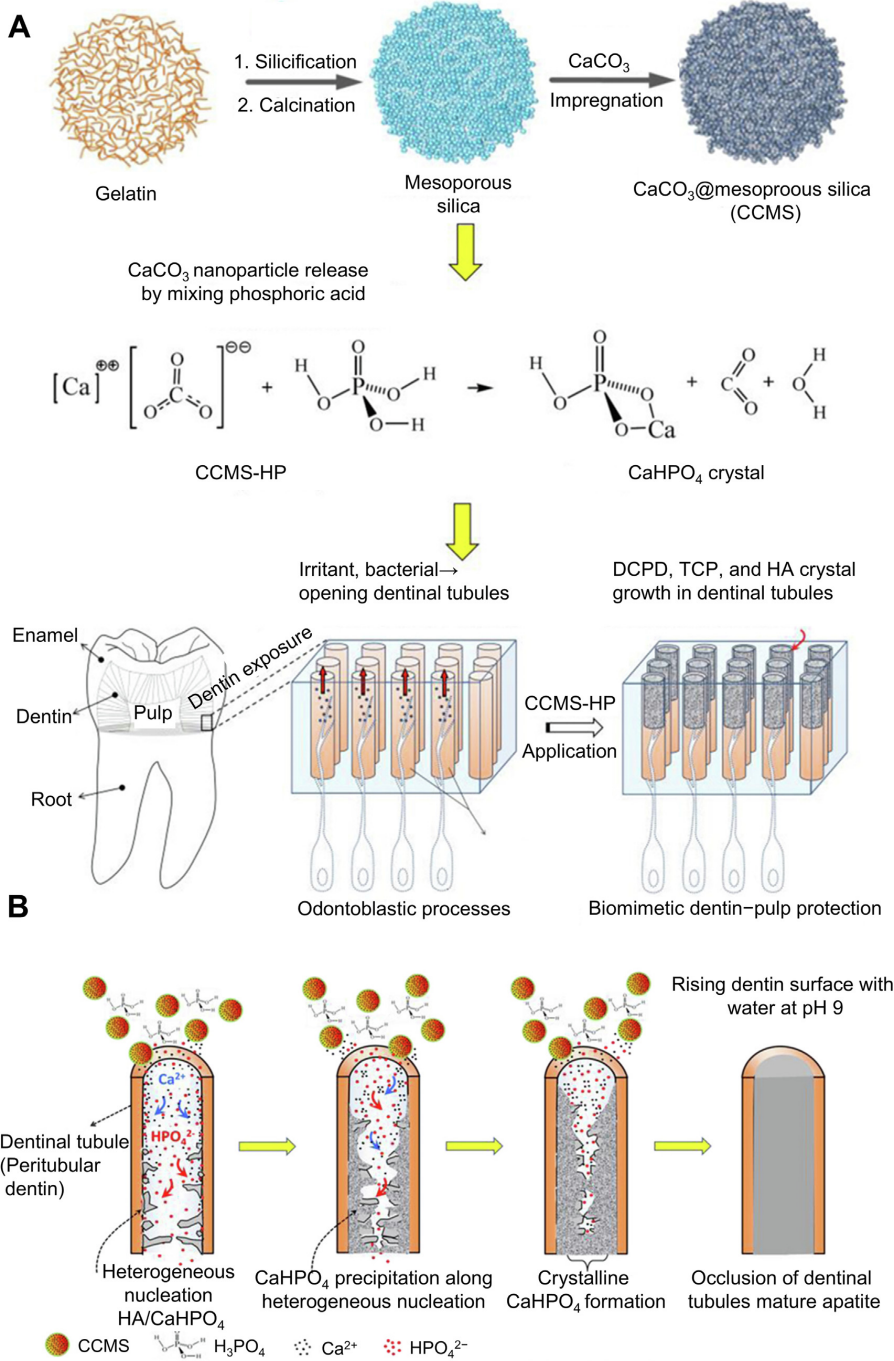
Application of ion precipitation method in relieve DH. (A) The synthetic pathway for CCMS and CCMS-HP was presented alongside a mechanism of CCMS-HP for biomimetic dentin-pulp protection. (B) Mechanism of crystallization process in a dentinal tubule. Reproduced with permission [24]. Copyright © 2014, American Chemical Society.

In addition, Chiang *et al.* developed the gelatin-templated mesoporous silica composite (CCMS), which incorporates nanoscale calcium carbonate (CaCO_3_) within its porous architecture. Upon reaction with 30 % phosphoric acid at a stoichiometric Ca/P ratio, the system releases bioactive ions (Ca^2+^, PO_4_^3−^, and HPO_4_^2−^) that diffuse into dentinal tubules and precipitate as dicalcium phosphate dihydrate (DCPD), tricalcium phosphate (TCP) and HA (Fig. 1A). Thus, the dentinal tubules can be effectively sealed by the resulting biomimetic crystalline precipitate. First, the mineralization process initiates when PO_4_^3−^ from H_3_PO_4_ solubilizes Ca^2+^ from the CCMS composite through an acid-base reaction. These liberated Ca^2+^ then diffuse into dentinal tubules, where they nucleate with available phosphate species to form amorphous calcium phosphate (CaP) precursors that subsequently mature into crystalline CaP deposits (Fig. 1B). These mesoporous biomaterials have the potential to be used as catalysts and carriers for dental complex tissue repair or regeneration [24].

**Fig. 2:**
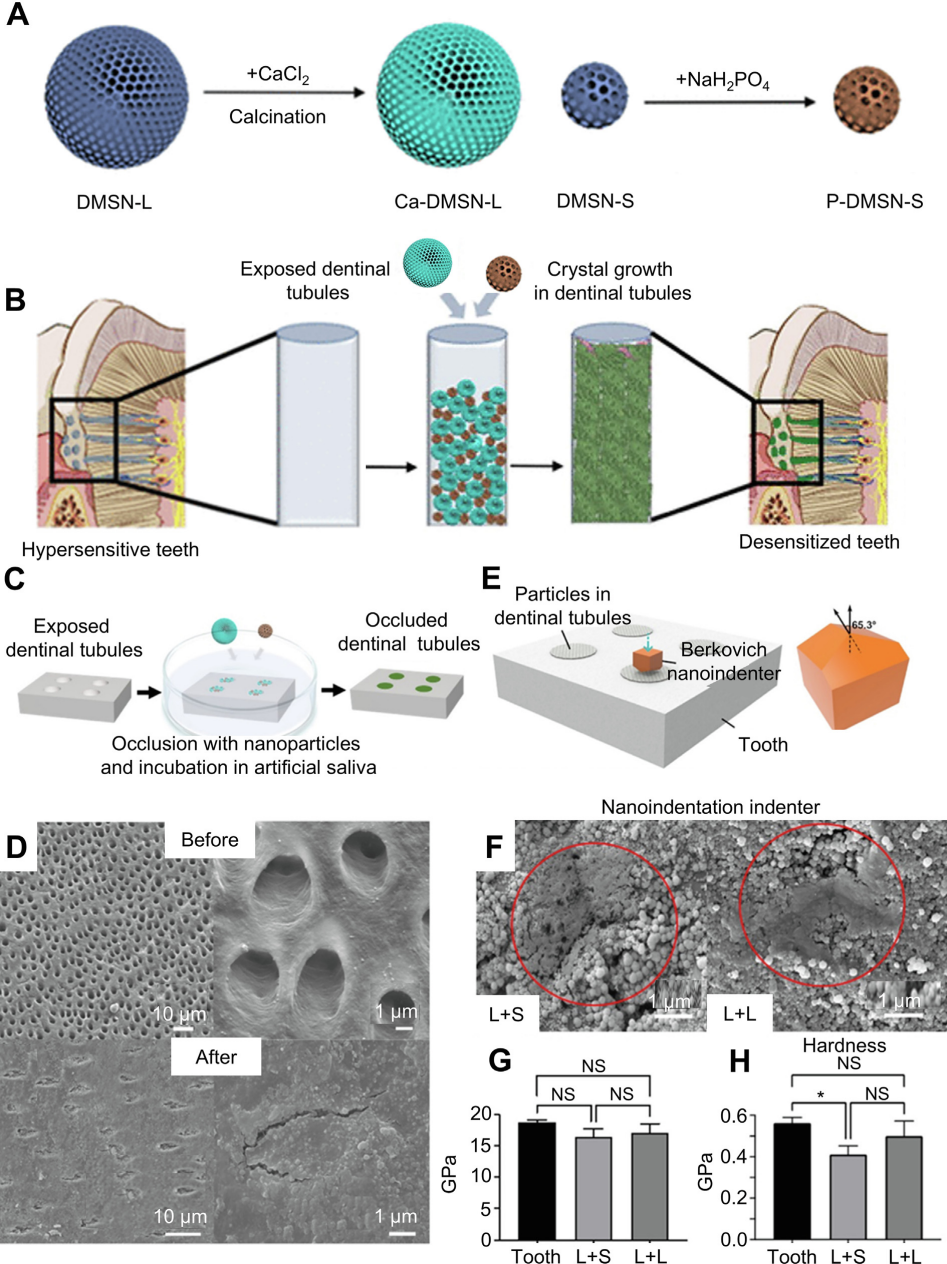
Application two different size particles to more tightly closed dentinal tubules. (A) Synthetic process for Ca-DMSN-L and P-DMSN-S. (B) Dentin tubule occlusion by inducing rapid growth of HA from a slurry of Ca-DMSN-L and P-DMSN-S. (C) Process of dentinal tubule occlusion with combo materials. (D) Cross-sectional views of dentin tubules before and after treatment. (E) Nanoindentation of dentin disks. (F) Nanoindentation of occluded dentinal tubules in L+S and L+L groups. The red circle highlights the indenter trace. The mechanical properties of occluded dentinal tubules were characterized by the reduced modulus (G) and hardness (H). All statistical data are represented as mean ± standard deviation (SD; *n* = 3; NS, no significant, *P < 0.05). Reproduced with permission [62]. Copyright © 2021, American Chemical Society.

Furthermore, scientists have developed a hierarchical desensitizing platform that combines large-scale calcium-doped mesoporous silica nanoparticles (Ca-DMSN-L) and nanoscale phosphate-doped silica nanoparticles (P-DMSN-S), which were prepared and mixed (L+S) (Fig. 2A,B). Topical application of the composite nanomaterial paste achieves rapid and complete dentinal tubule occlusion through a hierarchical desensitizing platform. Upon exposure to artificial saliva (3 × 1 min cycles), the material induces immediate hydroxyapatite (HA) nucleation and growth, forming a dense, mineralized barrier (Fig. 2C). Dentinal tubules in the L+S and L+L groups were completely occluded after treatment, as demonstrated in Fig. 2D. Nanoindentation of dentin discs nanoindentation SEM images of L+S and L+L indentation are shown in Fig. 2E,F. The red circle highlighted the characteristic indentation location. The nanoindentation data demonstrate that the mineral-occluded tubules in both treatment groups exhibited elastic modulus equivalent to native peritubular dentin but reduced hardness compared to peritubular dentin (Fig. 2G,H). Materials that seal dentinal tubules are highly resistant to acid and wear, indicating their potential for DH treatment [62].

### Nerve desensitization

The dental pulp is lined with a layer of highly specialized cells known as odontoblasts. *In vivo* studies have shown that sodium channels are expressed in odontoblasts. AnkyrinG colocalized with β2, suggesting a signal transduction link between axons and odontoblasts, which function as sensing cells that initiate the transmission of toothache [63]. Recent studies have identified TREK-1 and TRPV1/TRPA1 as essential mechanical channels for toothache perception [64]. External stimuli, such as cold, heat, and acidity, activate TRP channels in dentin-pulp afferent (DPA) fibers and odontoblasts. This activation releases ATP and glutamate, further stimulating the DPAs and contributing to tooth pain [65]. When tubule occlusion and neuronal communication are blocked, protecting the pulp nerves from external triggers, these channels become less active, which helps alleviate pain.

Clinical studies have demonstrated that dentifrices incorporating K salts, *e.g.*, potassium nitrate (KNO_3_) and potassium citrate (K_3_Cit), effectively alleviated clinical symptoms of DH [[66], [67], [68]]. KNO_3_ solution reduced DH by depolarizing cell membrane of the nerve terminals [69]. The mechanism of stannous fluoride (SnF_2_) induces chemical precipitation of insoluble metallic compounds that occlude exposed dentinal tubules, ultimately inhibiting the excitation of nociceptive nerve terminals [46].

Lasers, such as GaAlAs (780, 830, and 900 nm) as well as Nd:YAG lasers, inhibit nerve fiber depolarization (Aδ and C fibers) and suppress the generation and transmission of pain signals [18,70,71]. The Nd:YAG laser reduces pain by blocking the Na^+^/K^+^ pump, changing how membranes respond to stimuli, and affecting sensory nerve function [72]. This analgesic effect explains the immediate relief of toothache in patients after laser treatment [73].

Polyquaternium−10 (PQ−10), a cationic cellulose polymer hydrogel, has been shown to rapidly infiltrate dentinal tubules and alter the charged microenvironments, reducing the cationic current and alleviating DH symptoms [16]. The cationic hydrogel blocking (CHB) effect reduces the ionic conductance of dentinal tubules by impeding the entry of cations into the hydrogel network, whereas the anionic hydrogel blocking (AHB) effect enhances the ionic conductance. Therefore, CHB reduces stimulation-induced ionic current intensity through dentinal tubules, which can be improved by AHB. Correspondingly, the amplitude of nerve action potentials in response to pressure, pH, and temperature stimuli was significantly lower after CHB and markedly higher after AHB. Clinical investigations have shown that CHB significantly reduces patient pain. However, further research is needed to evaluate the duration of the curative effect of these charged materials.

### Protein precipitation

Dentin, like bone tissue, constitutes an extracellular matrix secreted by odontoblasts, which includes a variety of proteins, such as type I collagen (Col I). Gluma, a desensitizing agent, is a formulation that consists of glutaraldehyde combined with hydroxyethyl methacrylate (HEMA). Glutaraldehyde interacts with proteins in the dentinal tubules, leading to protein precipitation and subsequent blockage, effectively desensitizing the tooth HEMA, which is soluble in the dentinal tubular fluid, facilitates the penetration of glutaraldehyde into dentinal tubules, thereby diminishing their permeability. This forms a diaphragm that isolates bacteria and shields the pulp from external irritants. While glutaraldehyde-based desensitizers have shown effectiveness, limited studies have evaluated the biocompatibility, and findings suggest that glutaraldehyde remains cytotoxic under certain conditions. Silver diamine fluoride (SDF) is also an effective desensitizing agent through F-enhancing remineralization, forming fluor hydroxyapatite crystals to occlude dentinal tubules [[74], [75], [76], [77]]. In addition, silver ion (Ag^+^) denature proteins, leading to their aggregation in the dentinal tubules and relieving the symptoms of DH. While SDF's immediate occlusive effects are well-established, its long-term efficacy depends on continued protein precipitation.

## Synergistic occlusion of dentinal tubules

The primary mechanisms for relieving DH are physical and chemical strategies for occluding dentinal tubules. Desensitizers physically seal dentinal tubules by depositing crystals. However, complex oral microenvironments significantly limit their long-term efficacy, resulting in DH recurrence. This can be caused by factors, such as brushing the teeth or consuming acidic foods like citrus [73]. Clinical trials have also shown promising results for laser treatment of DH. Low-level power lasers, also called “soft lasers”, attenuate action potential propagation in intradental A−δ fibers and reduce transient receptor potential (TRP) channel sensitivity, preventing toothache from reaching the central nervous system by inhibiting depolarization [78]. However, their effectiveness seems to be poor with more severe DH. Therefore, a synergistic combination of both strategies may yield superior clinical outcomes by simultaneously sealing tubules and modulating pain perception.

### Laser combined desensitizing agents

In 1935, Grossman reported several relevant criteria for treating DH. The treatment must be rapid, long-term, easy to apply, not irritating the pulp, non-causing toothache, and not staining the tooth, and consistently effective [79]. The primary clinical interventions for managing DH typically involve the application of desensitizing agents, such as glass ionomer cement, dental adhesives, or specialized kinds of toothpaste containing active ingredients that occlude exposed dentinal tubules. The therapeutic effect is achieved through Ca and P deposition on dentin surface, sealing dentinal tubules. However, patients should be advised to use minimal water and avoid rinsing immediately after brushing, as this dilutes and washes away the active agent, thereby reducing its effectiveness [80].

Laser alleviates DH by providing direct analgesia to nerves, interrupting the depolarization of fibers, or inducing dentin re-crystallization, which mechanically seals the exposed dentinal tubules. The pain-reducing capacity of laser treatment exhibits limited duration in DH management, and after this effect diminishes, only the sealing mechanism remains, similar to that of desensitizing agents. In addition, given the narrow sizes of laser fibers, certain dentinal tubules may not receive adequate treatment [73,81]. Therefore, to achieve better desensitization, more researchers have demonstrated that the rapid and durable sealing of dentinal tubules can be achieved through the synergistic application of physical and chemical treatments. The combined therapy with GLUMA bonding and the 660 nm diode laser (0.09) with a lower visual analogue scale (VAS) than GLUMA bonding alone (0.78) after 30 days [82]. It appears that chemical agents initiate the mineralization process, which is then reinforced by a physical intervention to seal the dentinal tubules.

**Fig. 3:**
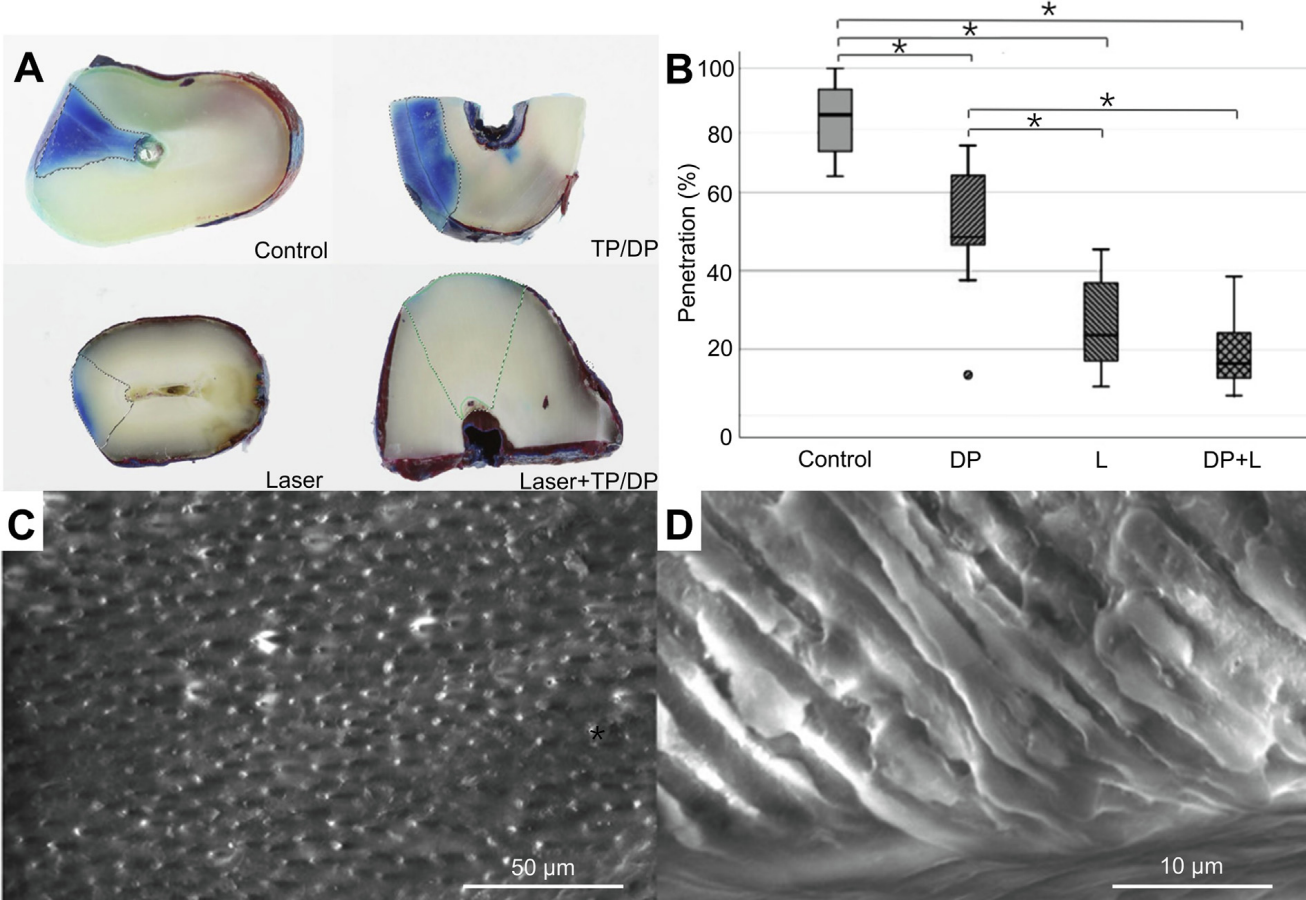
A combination treatment of laser irradiation and desensitizing agents in blocking dentinal tubules [83]. (A) Images depicting infiltration of dye into dentin. (B) Boxplot illustrating penetration depths for control group (Control), desensitizer paste group (DP), laser group (L), and combined desensitizer paste plus laser group (DP+L). (C,D) SEM images of cross-sectional (C) and longitudinal views (D) of dentin treated with combination of desensitizer paste and laser (DP+L). All statistical data are represented as mean ± SD (*n* = 10; NS, no significant, *P < 0.001).

Clinical studies demonstrate superior dentinal tubule occlusion when combining CO_2_ laser with tetracalcium phosphate/dicalcium phosphate (TeCP/DCP) anhydrous systems [83]. A total of 48 dental specimens were divided into four groups to perform dye penetration experiments. While control samples exhibited dye-deep penetration, DP-treated specimens showed reduced infiltration, and the L group displayed moderate restriction (23.7 %). Notably, the DP+L combination therapy achieved the most potent inhibition, with minimal penetration observed (16.5 %) (Fig. 3A), further supported by quantitative analysis (Fig. 3B). SEM images of the DP+L group revealed predominantly complete dentin tubule occlusion (Fig. 3C,D), confirming the enhanced sealing efficacy of combined treatment. The desensitizer TP/DP can spontaneously be converted in hydrated conditions to HA [84]. The photo-thermal energy generated by CO_2_ laser induces an instantaneous phase transformation in dentin minerals, converting their amorphous matrix into crystalline apatite structures [85,86]. The observed efficacy stems from a sequential process, initial physical sealing *via* DP deposition, followed by laser-induced fusion of DP with dentin matrix through a CO_2_ laser. Thus, clinical evidence suggests that combined therapy utilizing laser irradiation alongside topical desensitizing agents may represent a superior therapeutic approach for dentin hypersensitivity management.

Bioactive ceramic materials have been successfully implemented across various dental fields, including prosthetic rehabilitation, tissue regeneration, and direct restorative applications. Within restorative dentistry, these biomaterials demonstrate particular efficacy when used concomitantly with laser therapy for DH management [71]. Clinical studies confirm that combined BAG-laser treatment achieves superior dentinal tubule occlusion while simultaneously inducing sustained bioactive stimulation of pulpal stem cell activity [87]. K-doped bioactive glass (K-BAG) also serves as a dual-treatment strategy for DH, combining mechanical occlusion with neural desensitization. The material's therapeutic efficacy stems from its ability to simultaneously achieve complete physical blockage of dentinal tubules through nano-sized particle deposition while gradually releasing potassium ion (K^+^). This sustained ion release maintains elevated extracellular K^+^ level near interdental nerve, leading to membrane hyperpolarization and reduced neuronal excitability [88]. *In vitro* investigations conducted since 2005 have also demonstrated that combining this method with BAG improves clinical outcomes. However, the combination of laser and BAG has not yet shown sufficient effectiveness to advance to clinical trials [89].

### Biomimetic mineralization

The duration of desensitization is often limited due to friction during chewing and the erosive effects of acidic beverages like carbonated drinks. Therefore, it is crucial to identify a material that maintains the desensitization effect over time. Biomimetic mineralization, which mimics the natural biological mineralization process, is a promising approach. This method utilizes Ca and P clusters to create a mineralization front, promoting the directional epitaxial growth of enamel crystal and replicating the intricate structure of natural enamel [90,91]. This strategy not only improves the durability of tooth restoration but also reconstructs complex structure of the tooth to achieve a better restoration effect. It has significant application value for the treatment of DH.

Advances in chemistry have transformed dentin remineralization strategies from traditional thermodynamic deposition methods to biomimetic approaches that more accurately replicate biological mineralization processes. This paradigm shift enables precise restoration of both the hierarchical microstructure and biomechanical properties of demineralized dentin. A biomimetic mineralization model was developed by Ling *et al.*, creating optimal conditions for dentin-pulp complex regeneration, which effectively occluded dentinal tubules on acid-etched dentin surface, regenerating enamel-like tissue containing fluorinated HA crystals. The newly formed enamel-like structure demonstrated higher microhardness values than the demineralized dentin substrate, indicating successful biomimetic remineralization [92].

**Fig. 4:**
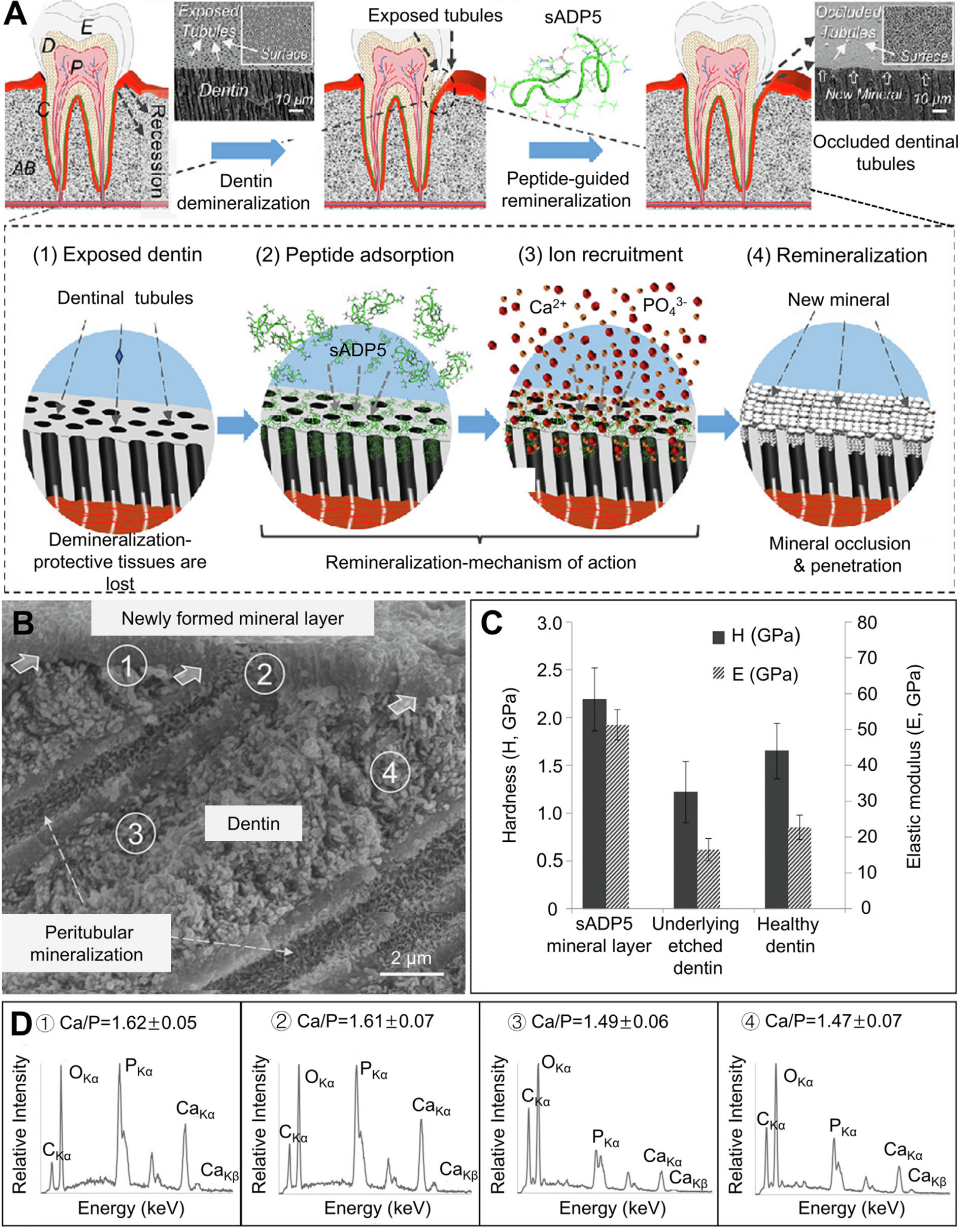
Peptide-guided biomimetic remineralization of exposed human dentin [93]. (A) Remineralization cycle *in vitro*. (B) Cross-section teeth specimens. (C) The mechanical properties were assessed *via* nanoindentation at various spatial locations on cross-sectioned samples, which revealed sound dentin, etched tooth, and newly formed mineral. (D) The elemental composition was analyzed by collecting EDXS spectra from four distinct regions labeled 1 through 4. The displayed Ca/P ratios were calculated using the Kα peaks of Ca and P from these spectra. Abbreviations: AB, alveolar bone; C, cementum; D, dentin; E, enamel; P, pulp; sADP5, shortened amelogenin-derived peptide 5.

Yucesoy *et al.* demonstrated successful human dentin restoration using sADP5 peptide (15 amino acids), which directed Ca and P remineralization with enhanced kinetics and confirmed both superficial mineral deposition and intratubular penetration depth (Fig. 4A,B). A single round of peptide-guided remineralization treatment resulted in a 0.8 ± 0.3 μm-thick continuous mineral layer on the dentin surface [93]. Simultaneously, Ca/P stoichiometry of the newly formed surface mineral demonstrated compositional equivalence to hydroxyapatite (Fig. 4D). Quantitative measurements showed site-specific variations in mineral composition, with Spectra 1−2 maintaining near-ideal Ca/P ratios (∼1.67) comparable to reference apatites, whereas Spectra 3−4 displayed significantly lower ratios. These systematic differences directly correlate with the controlled demineralization pretreatment of dentin surface. During this process, Ca diffusion profiles showed preferential accumulation within a 10 μm subsurface zone. Mechanical testing indicated distinct hardness and elastic modulus across all examined regions, with the newly formed mineral layer displaying stiffer than dentin, demonstrating optimal mechanical characteristics (Fig. 4C). Structural characterization reveals that the newly formed mineral phase functionally emulates the graded tissue interface observed in native dentin−enamel junctions, achieving comparable mechanical interlocking. During biomimetic mineralization, the amelogenin-derived peptide sADP5 is a bioactive template that initiates and directs the primary remineralization process. Mineral deposition on the dentin surface and subsequent intratubular penetration effectively occlude exposed dentinal tubules through biomimetic mineralization and restore natural protective effect of the tooth.

**Fig. 5:**
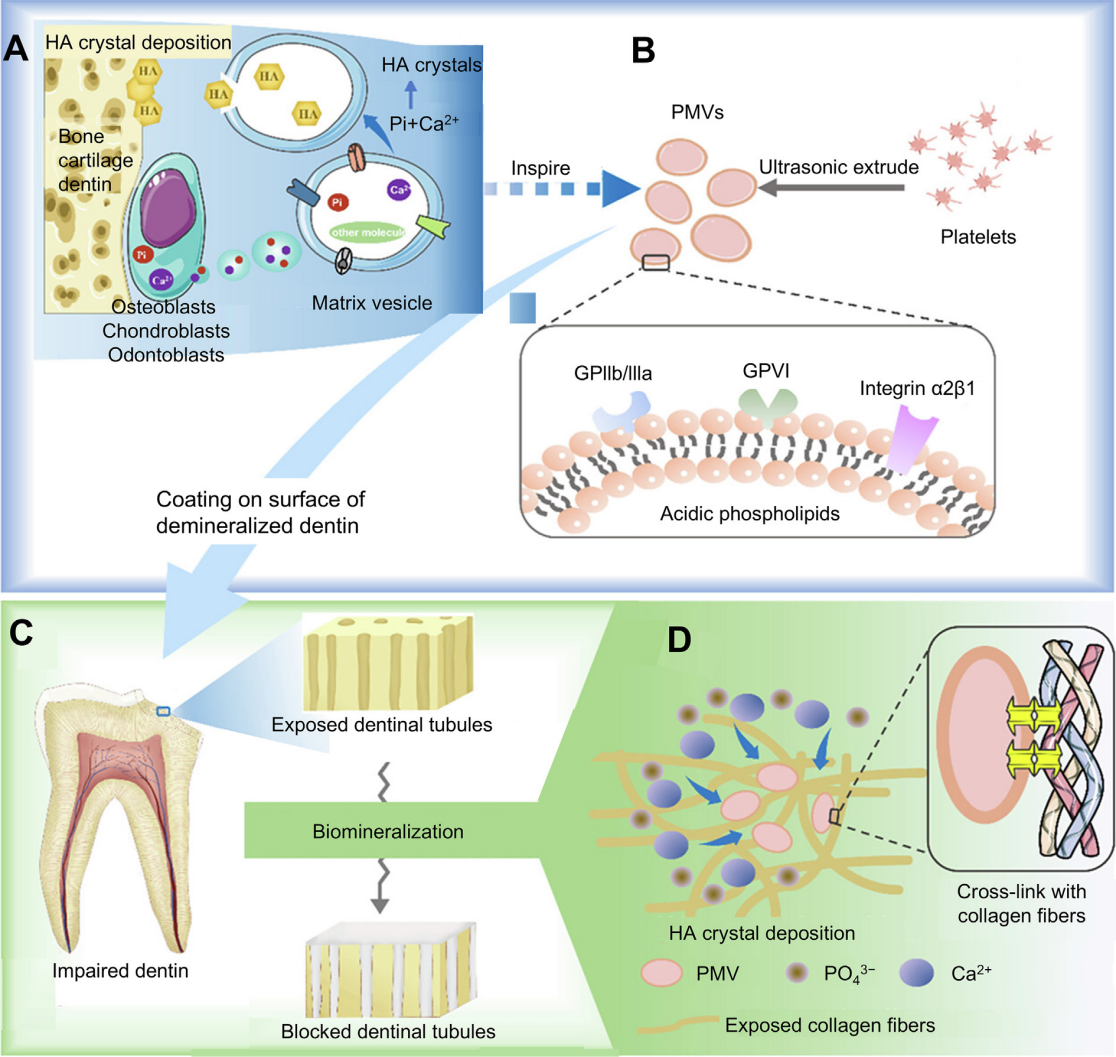
PMVs mediate dentin regeneration. (A) Engineering artificial MVs by reconstructing platelet membrane components, preserving native MVs key structural and functional characteristics. (B) After isolating the membrane from platelets, it was transformed into vesicles *via* ultrasound and extrusion processes, ensuring the complete preservation of membrane proteins. (C) PMVs were applied to compromised dentin, where they facilitated biomineralization within the deep dentinal tubules, ultimately leading to effective dentin repair. (D) The biomineralization process involved PMVs interacting with demineralized Col fibers, leveraging the vesicle structure, membrane proteins, and acidic phospholipids to promote mineralization. Reproduced with permission [94]. Copyright © 2023, American Chemical Society.

Furthermore, researchers have developed platelet membrane vesicles (PMVs) replicating key features of native matrix vesicles. These PMVs demonstrate excellent biocompatibility, high colloidal stability, and strong dentin affinity. The surface-exposed acidic phospholipids on nanovesicles serve as nucleation sites that selectively recruit Ca^2+^ and PO_4_^3−^ from the local microenvironments, facilitating spatially controlled biomineralization (Fig. 5A,B). Their nanostructure ensures smooth penetration into dentinal tubules and provides a good three-dimensional (3D) space for mineralization similar to platelet membrane vesicles. At the same time, the biocompatible PMV safeguards dentin's organic matrix and directs mineral deposition within tubules, culminating in structurally stable occlusion and functional dentin rehabilitation (Fig. 5C,D). The 3D analysis confirmed PMV-mediated mineral formation within deep dentinal tubules to depths exceeding 60 μm and demineralized surface. This innovative application of cellular vesicles for structural biomineralization opens new avenues in restorative dentistry through biomimetic tissue engineering [94]. Hard tissue regeneration is the fundamental and challenging solution in relieving DH. Numerous studies have systematically explored to identify the ideal method of repairing damaged hard tissue, showing promise for clinical applications and holding potential for further development [95].

Although *in vitro* studies have demonstrated the synergistic effects of combined therapies in occluding dentinal tubules or reducing permeability, evidence from *in vivo* clinical studies supporting their efficacy and clinical translatability remains limited. This limitation significantly hinders the widespread adoption of combined therapies in clinical practice.

## Conclusion and perspectives

The treatment of DH remains a significant challenge for dental professionals. The key to effectively managing this condition is the rapid and reliable sealing of dentinal tubules to ensure patient comfort. A comprehensive understanding of the pathogenesis of DH is essential, as it forms the basis for developing novel treatment approaches. Numerous studies have explored various physical, chemical, and combined approaches to seal dentinal tubules and alleviate DH. Nonetheless, there is still room for improvement in the speed, efficacy, and durability of these treatments.

Physical occlusion of the dentinal tubules includes laser desensitization and invasive restoration. Laser has demonstrated rapid efficacy in treating DH by diminishing the water content in dentin through thermal effects, potentially enhancing the physical properties and contributing to stabilization of the HA. However, the heat generated by laser poses a risk of inducing cracks in the dental hard tissues, potentially causing irreversible damage to dental structures and even affecting the dental pulp. Laser is often combined with desensitizing agents to mitigate this risk and prevent dentin cracking. Desensitizing agents form protective coatings on dentin surface while simultaneously releasing ions that diffuse into the dentinal tubule, precipitating from a mechanical occlusion and providing adhesive force. Invasive restoration treatment is generally a solution when non-surgical treatment is ineffective. However, it requires a higher skill level from clinicians and incurs a higher cost.

Chemical occlusion of the dentinal tubules involves ion precipitation, nerve desensitization, and protein precipitation. Many desensitizing compounds work by forming mineralized deposits within exposed dentinal tubules, which block the tubules, promote dentin remineralization, and reduce nerve excitability to alleviate toothache. These agents also interact with the proteins in the dentinal tubules to cause protein precipitation and seal the tubules. Given the complex oral microenvironments exposed to various stimuli, such as temperature changes and the mechanical forces from chewing, these ions gradually deplete and need continuous replenishment of ions to maintain function. As a result, the symptoms of DH re-emerge.

Synergistic occlusion of dentinal tubules, including biomimetic mineralization, laser-assisted desensitizing agents, and dual desensitizers with complementary mechanisms, has demonstrated highly effective clinical results. When laser is combined with desensitizers, these desensitizers release ions that deposit on the dentin surface, helping to repair hard tissue fractures caused by the high heat of laser. It is necessary to develop durable and acid-resistant materials to significantly enhance the immediate and long-term efficacy of DH treatment, especially when combined with laser therapy. Concurrently, remineralization mimicking the natural mineralization process, promoting long-term occlusion of dentinal tubules without artificial blockage, and forming stable mineral layers to reduce the risk of recurrence have become a cornerstone of DH treatment. Unlike laser or adhesives, remineralization integrates seamlessly with the tooth structure, avoiding thermal or chemical damage. Furthermore, remineralization replenishes lost mineral components (*e.g.*, Ca and P) within the tooth structure, improving its microhardness and acid resistance. Since remineralization is a slow and progressive process, its therapeutic efficacy inherently depends on long-term patient compliance.

Overall, treatment methods that seal dentinal tubules and reduce neural sensitivity are effective measures for treating DH. It is necessary to continue developing new approaches to seal dentinal tubules or block nerve transmission. For example, materials with better biocompatibility and improved efficacy in sealing dentinal tubules hold promise. These include advanced forms of CaP, BAG, and other mineralizing agents that promote natural remineralization. Nanoparticles and nanocoatings may be designed to penetrate dentinal tubules more effectively, providing a long-lasting barrier against stimuli and reducing sensitivity.

Additionally, biotechnological methods, such as gene therapy or tissue engineering, offer new ways to address the root causes of DH, potentially leading to regenerative treatment that restores dentin structure and function. Developing dental devices for home use, such as varnishes or gels that release active ingredients over time, provides a convenient and effective solution for patients to alleviate DH. Research into stem cell therapies and other regenerative approaches may ultimately lead to treatments that repair or regenerate dentin and pulp tissue, addressing the underlying cause of DH.

## Compliance with ethics requirements

This article does not contain any studies with human or animal subjects.

## Declaration of competing interest

The authors declare that they have no known competing financial interests or personal relationships that could have appeared to influence the work reported in this paper.
